# Breast cancer surgery during the Covid-19 pandemic: a monocentre experience from the Regina Elena National Cancer Institute of Rome

**DOI:** 10.1186/s13046-020-01683-y

**Published:** 2020-08-27

**Authors:** Fabio Pelle, Sonia Cappelli, Franco Graziano, Loredana Piarulli, Flavia Cavicchi, Domenico Magagnano, Assunta De Luca, Roy De Vita, Marcello Pozzi, Maurizio Costantini, Antonio Varanese, Massimo Panimolle, Pietro Paolo Gullo, Maddalena Barba, Patrizia Vici, Enrico Vizza, Francesco Cognetti, Giuseppe Sanguineti, Elena Saracca, Gennaro Ciliberto, Claudio Botti

**Affiliations:** 1 Department of Surgery, Division of Breast Surgery, IRCCS Regina Elena National Cancer Institute, Via Elio Chianesi 53, 00144 Rome, Italy; 2 Quality and Risk Management, Medical Direction, IRCCS Regina Elena National Cancer Institute, Via Elio Chianesi 53, 00144 Rome, Italy; 3 Department of Surgery, Division of Plastic and Reconstructive Surgery, IRCCS Regina Elena National Cancer Institute, Via Elio Chianesi 53, 00144 Rome, Italy; 4 Division of Medical Oncology 2, IRCCS Regina Elena National Cancer Institute, Via Elio Chianesi 53, 00144 Rome, Italy; 5 Unit of Gynecologic Oncology, Department of Experimental Clinical Oncology, IRCCS Regina Elena National Cancer Institute, Via Elio Chianesi 53, 00144 Rome, Italy; 6 Division of Medical Oncology 1, IRCCS Regina Elena National Cancer Institute, Via Elio Chianesi 53, 00144 Rome, Italy; 7 Department of Radiation Oncology, IRCCS Regina Elena National Cancer Institute, Via Elio Chianesi 53, 00144 Rome, Italy; 8 Department of Radiology, IRCCS Regina Elena National Cancer Institute, Via Elio Chianesi 53, 00144 Rome, Italy; 9 Scientific Direction, Regina Elena National Cancer Institute, Via Elio Chianesi 53, 00144 Rome, Italy

**Keywords:** Covid-19 pandemic; elective breast cancer surgery;, safety;, Covid-19 protective cancer hubs

## Abstract

The Covid-19 pandemic has challenged hard the national health systems worldwide. According to the national policy issued in March 2020 in response to the evolving Covid-19 pandemic, several hospitals were re-configured as Covid-19 centers and elective surgery procedures were rescheduled according to the most recent recommendations. In addition, Covid-19 protected cancer hubs were established, including the Regina Elena National Cancer Institute of Rome, Central Italy. At our Institute, the Breast Surgery Department continued working under the sign of a multidisciplinary approach. The number of professional figures involved in case evaluation was reduced to a minimum and interactions took place in the full respect of the required safety measures. Treatments for benign disease, pure prophylactic surgery and elective reconstructive procedures were all postponed and priority was assigned to the histologically-proven malignant breast tumors and highly suspicious lesions. From March 15th though April 30th 2020, we treated a total of 79 patients. This number is fully consistent with the average quantitative standards reached by our Department under ordinary circumstances. Patients were mostly discharged the day after surgery and none was readmitted due to surgery-related late complications. More generally, post-operative complications rates were unexpectedly low, particularly in light of the relatively high number of reconstructive procedures performed in this emergency situation. A strict follow up was performed based on the close contact with the surgical staff by telephone, messaging apps and telemedicine.

Patients ascertainment for their Covid-19 status prior to hospital admission and hospital discharge allowed to maintain the “no-Covid-19” status at our Institution. In addition, during the aforementioned time window, none of the care providers developed SARS-CoV-2 infection or disease, as shown by the results of anti-SARS-CoV-2 immunoglobulin M and G profiling. In conclusions, elective breast cancer surgery procedures were successfully performed in a lockdown situation due to a novel viral pandemic. The well-coordinated regional and hospital efforts in terms of medical resource re-allocation and definition of clinical priorities allowed to maintain high quality standards of breast cancer care while ensuring safety to the cancer patients and care providers involved.

## Background

On the 20th of February 2020, in Italy, the first case of acute interstitial pneumonia related to an infection by severe acute respiratory syndrome coronavirus-2 (SARS-CoV-2) was detected. Within few weeks, the number of cases of SARS-CoV-2 infection and related disease (Covid-19) increased exponentially until the 21st of March, when a slow and constant decline began. The World Health Organization (WHO) declared the Covid-19 outbreak as a pandemic on the 11th of March 2020 [1–3]. In response to the evolving Covid-19 pandemic, a nationwide lockdown with restrictive measures was issued. In Italy, several hospitals were re-configured as Covid-19 centers and elective surgery procedures were postponed according to the most recent recommendations [4]. In order to ensure adequate treatments to cancer patients, Covid-19 protected cancer hubs were established in the Lazio Region, Central Italy.

Early reports from China have conveyed a quite alarming message concerning an increased risk of SARS-CoV-2 infection and disease with particularly unfavorable outcomes in cancer patients. Subsequent work has clarified the need of interpreting this evidence with caution in light of the restricted sample size, heterogeneity of the study population, bias and confounding which may all concur to limit the generalizability of these findings to the entire cancer patient population, at a national and international level [5, 6]. However, in the Covid-19 era, the risks related to elective surgical procedures need to be newly explored relatively to patients’ status concerning SARS-CoV-2 infection and disease. Indeed, surgery in an unrecognized Covid-19 positive patient may have dramatically detrimental effects [7]. In specific regard to cancer patients, to ensure continuation of care delivery while preserving safety from SARS-CoV-2 infection and disease, screening of asymptomatic patients and care providers is of pivotal importance, along with the establishment and implementation of preventive measures to avoid contact with potential Covid-19 carriers [8].

The Breast Surgery of the Regina Elena Cancer Institute of Rome is an OECI-accredited tertiary referral facility, which includes two Surgical Divisions, i.e., the Breast and Plastic Reconstructive Surgeries, that overall perform about 600 procedures for primary breast cancer yearly; it is located in a wing of the main hospital building, apart from other surgical Divisions and allocates twenty-four adjustable beds in twelve rooms of twenty square meters with bathroom. At the time the national lockdown was declared, our Institution was identified as a regional oncologic hub. We herein report on the experience matured at the Department of Breast Surgery during the lockdown issued in response to the Covid-19 pandemic. Results are reported and discussed concerning the quali-quantitative standards reached under ordinary circumstances, as well as in light of key safety issues and inherently adopted measures.

## Main text

All the pre-scheduled treatments for benign disease, pure prophylactic surgery as well as elective reconstructive procedures were postponed, and priority was given to the histologically-proven malignant breast tumors and highly suspicious lesions defined as such on the basis of tru-cut biopsies and/or clinical-radiological findings. Ad hoc internal standardized operative procedures were developed and implemented by the institutional risk management team, in full agreement with the guidelines released from the Italian Minister of Health and Health Department of the Lazio Region, which were constantly reviewed and shared through the intranet Institutional web pages. The following safety measures were established:

1. Triage procedures were applied to each cancer patient prior to hospital admission, independently on the specific regimen, e.g., ordinary vs ambulatory. These procedures were repeated prior to hospital discharge to unveil unnoticed hospital exposures. In both the cases, specifically trained personnel administered an ad hoc questionnaire focused on the recent occurrence of the most common Covid 19-related symptoms and contacts with people from the “red zones”. In addition, prior to their admittance to the hospital building, patients’ body temperature was detected by thermo scanners
2. Nasopharyngeal swab 48 h before the scheduled surgery
3. Limited access to the pre-hospitalization area
4. Patients staying in single rooms and wearing face masks
5. No relatives admitted to the ward. In the case of not self-sufficient patients, only one relative with face mask and negative triage/swab was allowed
6. Adequate personal protective equipment, including disposable surgical masks, gloves, gowns and face shields, when deemed necessary
7. Personal hand hygiene before and after contact with patients
8. Daily assessment of body temperature for patients and staff
9. Reduced crowding in operative room and adequate distance in the awakening area
10. Lowered amount of aerosol during the operative procedures, e.g., lowest effective power of electrocautery or other energy devices to decrease smoke, extra precaution during creation of pneumoperitoneum and gas deflation in case of concomitant laparoscopic bilateral salpingo-oophorectomy in case of BRCA1/2 mutated breast cancer cases.

We continued ensuring multidisciplinary evaluations, which involved one single general surgeon, oncologist, radiotherapist, and plastic surgeon all maintaining adequate social distance and wearing masks. Seven patients had their programmed surgery delayed because of the onset of clinical symptoms (mild fever in 3 cases, unilateral conjunctivitis in 1 case), presumably positive swab (2 cases), contact with suspected Covid-19 positive relatives (1 case). All these cases were rescheduled after 15 days following symptoms resolution and 2 consecutive negative swabs. A negative swab was not considered a safety parameter in presence of suspicious symptoms. From March 15th through April 30th, we treated a total of 79 patients (Table 1). In brief, breast conserving surgery was performed in 29 patients. Reconstructive surgery was performed in 48 patients (60.7%). Digital mammography was routinely performed and if the subcutaneous tissue thickness was less than 1.5 cm, retropectoral implants positioning was preferred. One stage breast reconstruction implant based and pre-pec positioning was the preferred option (16, 33.3%) following the preliminary good results obtained with this technique [9]. Tissue expanders (TE) were placed in 14 cases as a safe bridge to definitive reconstruction. Laparoscopic bilateral salpingo-oophorectomy concomitantly with bilateral mastectomy and immediate reconstruction were performed in 2 BRCA1 mutated patients. Two cases were classified as benign at final pathology. No patient developed postoperative respiratory symptoms. Postoperative fever was observed in 2 cases but resolved spontaneously after 48 h with negative chest CT scan. No early or late surgical complication were observed in this group of patients. Patients were mostly discharged the day after surgery. No patient was readmitted to the hospital due to surgery-related late complications. Patients were followed on an outpatient basis keeping a close contact with the surgical staff by telephone, messaging apps and telemedicine. No delays were observed in histological diagnosis and timing of adjuvant treatments. No surgeons or nurses developed SARS-CoV-2 infection or disease. As of the10^th^ of May 2020, all staff members have been tested for Anti-SARS-CoV-2 immunoglobulin M and G, and none resulted positive.

**Table 1:** Descriptive characteristics of the study participants (N: 79)

	Mean (range)
**Age (years)**	56.7 (34–89)
	**N (%)**
**Histology**	
**Benign**	1 (1.3)
**Malignant**	78 (98.7)
*Invasive lobular carcinoma*	15 (19.2)
*Invasive ductal carcinoma*	55 (70.5)
*Miscellaneus*^*a*^	4 (5.1)
*Unknown*^*b*^	4 (5.1)
Grading (G)*	
**G 1–2**	32 (41.0)
**G3**	40 (51.3)
**Unknown**^**c**^	6 (7.7)
Hormon receptor status*	
^**d**^**ER+/**^**e**^**PgR+**	57 (73.1)
**ER+/PgR-**	12 (15.4)
**ER−/PgR-**	6 (7.7)
**Unknown** ^**f**^	3 (3.8)
Her2 Status*	
**Positive**	12 (15.2)
**Surgical procedures**	
**Surgical procedures with curative intent**	79 (100)
*Simple excision/lumpectomy*	2 (2.5)
*Breast conserving surgery*	29 (36.7)
^*g*^*Mastectomy*	23 (29.1)
^*h*^*Nipple sparing mastectomy*	19 (24.0)
^*i*^*Skin reducing mastectomy*	6 (7.6)
**Surgical procedures with recostructive intent**	48 (60.7)
*Pre-pectoral DTI*^*j*^	16 (33.3)
*Sub-pectoral DTI*	4 (8.3)
*Pre-pectoral TE*^*k*^	1 (2.1)
*Sub-pectoral TE*	13 (27.1)
*Contralateral symmetrization*	4 (8.3)
Surgical procedures with prophilactic intent**	
*Concomitant bilateral oophorectomy*	2 (2.5)
**Neo-adjuvant chemotherapy**	
*Yes*	13 (16.7)
**Pathologic complete response**	
*Yes*	5 (38.5)
^**l**^**ASA risk score**	
*1–2*	60 (76.0)
*3–4*	19 (24.0)

Legend: ^a^Miscellaneous: Special hystologic types, e.g., cribiform carcinoma, mucous carcinoma, medullary carcinoma, squamous carcinoma, papillary carcinoma, apocrine carcinoma; ^**b**^ Cases with unknown histology include: a. one patients with pCR following neo-adjuvant chemotherapy; b. one patient with inflammatory mastitis; c. one patient who underwent breast biopsy with no residual breast cancer at the histological assessment of residual breast; d. one patient who underwent lumpectomy with no residual breast cancer at the histological assessment of residual breast; ^**c**^Cases with unknown grading include: a. 3 cases with diagnostic workup and surgical cancer excision out of the Regina Elena National Cancer Institute; b. one patients with pCR following neo-adjuvant chemotherapy; c. one case of carcinomatous mastitis; d. one case who underwent lumpectomy with no residual breast cancer at the histological assessment of residual breast; ^**d**^ ER: Estrogen Receptors; ^**e**^ PgR: Progesterone Receptors; ^**f**^ Hormonal Receptor Status was unknown in the following: a. one case with diagnostic workup and surgical cancer excision out of the Regina Elena National Cancer Institute**; b.**. one patients who achieved pCR following neo-adjuvant chemotherapy; c. one case of carcinomatous mastitis; * For these variables, percentages were computed over a total number of 78 patients, i.e., the overall number of patients with histologically-confirmed breast malignancy, with the only case of histologically-proven benign tumor being excluded; ** For this variable, percentage was computed over a total number of 79, i.e., the overall number of surgical procedures performed; ^g^ Of the 23 mastectomies, 4 were bilateral mastectomies; ^h^ Of the 19 nipple sparing mastectomies, 5 were bilateral; ^i^ Of the 6 skin reducing mastectomies, 3 were bilateral; ^j^DTI: Direct-to-Implant; ^k^ TE: Tissue Expander; ^l^ASA: Amercan Society of Anesthesiologists

This experience clearly shows that elective breast cancer surgery can be safely pursued even in a lockdown situation due to a novel viral pandemic. Thanks to well-coordinated regional and hospital efforts in terms of medical resource re-allocation and definition of clinical priorities, we were able to maintain high quality standards of breast cancer care while mitigating the backlog effect of elective surgical cases once the immediate Covid-19 threat was ebbed. The unexpected absence of increased rates of post-operative complications observed in light of the relative high number of reconstructive procedures performed in this emergency situation confirms what reported elsewhere [10]. It probably reflects the beneficial effect of the preventive measures adopted in terms of social distancing and hand hygiene, thus suggesting that new behavioral standards should be adopted in our (staff and patients) daily life in and outside the hospital.

Lastly, we were worried that a reiterated triage could be interpreted by our patients as barrier to their expectations of an expedite operation and induce them to conceal some information. On the contrary, all patients realized the importance of the procedures applied and helped us delineate the complex and rapidly evolving epidemiologic scenario, despite the danger of cancelling previously scheduled surgery.

## Conclusions

During the lockdown issued in Italy in response to the Covid-19 pandemic, the Breast Surgery Department of the Regina Elena National Cancer Institute continued working according to a multidisciplinary approach. Priority was given to the histologically-proven malignant breast tumors and highly suspicious lesions. From March 15th though April 30th 2020, 79 patients were treated, consistently with our average quali-quantitative standards. Hospitalization was mostly concluded the day after surgery. Post-operative complications rates were unexpectedly low. Telephone contacts, messaging apps and telemedicine allowed for a close follow up. The “no-Covid-19” status of our Institution was maintained. None of the care providers involved developed SARS-CoV-2 infection or disease. In brief, we maintained acceptable quali-quantitative standards of breast cancer care while ensuring safety to our cancer patients and care providers involved.

## Data Availability

Data will be made from the corresponding author on reasonable request.

## Abbreviations

Covid-19: Coronavirus disease 2019
CT: Computed tomography
OECI: Organization European Cancer Institutes
SARS-CoV-2: Severe acute respiratory syndrome coronavirus-2
